# Building the Clinical Bridge to Advance Education, Research, and Practice Excellence

**DOI:** 10.1155/2012/826061

**Published:** 2012-03-22

**Authors:** Marilyn Svejda, Janet Goldberg, Maureen Belden, Kathleen Potempa, Margaret Calarco

**Affiliations:** ^1^Division of Health Promotion & Risk Reduction Programs, School of Nursing, University of Michigan, 400 North Ingalls, Ann Arbor, MI 48109-5482, USA; ^2^Division of Nursing Business & Health Systems Programs, and Office of Academic Affairs, School of Nursing, University of Michigan, 400 North Ingalls, Ann Arbor, MI 48109-5482, USA; ^3^Division of Acute, Critical & Long-Term Care Programs, School of Nursing, University of Michigan, 400 North Ingalls, Ann Arbor, MI 48109-5482, USA; ^4^Office of the Dean, School of Nursing, University of Michigan, 400 North Ingalls, Ann Arbor, MI 48109-5482, USA; ^5^Nursing Administration, University of Michigan Health System, 300 North Ingalls, Suite 5A04, Ann Arbor, MI 48109-5446, USA

## Abstract

The University of Michigan School of Nursing and the Health System partnered to develop an undergraduate clinical education model as part of a larger project to advance clinical education, practice, and scholarship with education serving as the clinical bridge that anchors all three areas. The clinical model includes clusters of clinical units as the clinical home for four years of a student's education, clinical instruction through team mentorship, clinical immersion, special skills preparation, and student portfolio. The model was examined during a one-year pilot with junior students. Stakeholders were largely positive. Findings showed that Clinical Faculty engaged in more role modeling of teaching strategies as Mentors assumed more direct teaching used more clinical reasoning strategies. Students reported increased confidence and competence in clinical care by being integrated into the team and the Mentor's assignment. Two new full time faculty roles in the Health System support education, practice, and research.

## 1. Introduction

Over the past several years, schools of nursing have been called upon to restructure education programs to better prepare graduates for increasingly complex and rapidly changing health care environments [1–3]. According to Benner and associates [2], nursing education programs must be redesigned to prepare nurses for new responsibilities and challenges in these health care environments. To accomplish this, the practice-education gap must be addressed by major shifts in both curricula and teaching methods [2]. The call to revise nursing education programs is paralleled by similar calls from other organizations including the Robert Wood Johnson Foundation (RWJF) [4] and the Institute of Medicine (IOM) [5]. The Institute of Medicine's 2001 report, *Crossing the Quality Chasm: A New Health System for the 21st Century, *recommended that leaders in health professions should develop strategies for “restructuring clinical education to be consistent with the principles of the 21st-century health system throughout the continuum of undergraduate, graduate, and continuing education for medical, nursing, and other professional training programs” (Recommendation 12, p. 208). The recent consensus report of the RWJF in collaboration with the IOM, “Initiative on the Future of Nursing: Leading Change, Advancing Health” [6], emphasizes that nurses should achieve higher levels of education and training through an improved education system that promotes seamless academic progression.

Recently, partnerships between schools of nursing and service settings in hospitals and other health care agencies led to the development of new approaches to improve clinical learning. The goal of these partnerships is to facilitate students' understanding of both the responsibilities of the nurse and the environments in which nurses work as they acquire clinical knowledge and proficiency necessary for competent practice. One common approach in hospital settings is the Dedicated Education Unit (DEU). Pioneered in Australia [7], DEUs are health care units either enhanced or developed through partnered commitments by clinical faculty and nursing staff to enrich student nursing clinical education. To help students complete learning objectives, an environment is created to provide optimal learning opportunities using a variety of teaching and learning strategies [7–13]. In the DEU model, education responsibilities may not be shared equally between the clinical faculty and clinical nursing staff. While clinical faculty are present to assist with student learning and model good teaching practices, the clinical staff assumes more responsibility for direct instruction of students assigned to them. Typically, the number of DEUs in any health care setting is small. Thus, not all students experience this educational concept during their program of study in nursing. Those not participating receive the more traditional model where the clinical faculty assumes nearly all responsibility for clinical instruction with less intensive involvement of staff nurses. In the traditional approach, clinical faculty and students often are viewed as “guests” on the units that host students as neither the faculty or students are fully integrated into unit functioning.

Outcomes of the DEU concept of clinical education for undergraduate nursing students are not extensive, and the concept continues to be explored. Extant findings [9, 10, 12, 13] document positive outcomes to this approach in clinical education. Collectively, outcomes show that students report feeling welcomed and supported by nursing staff, and included as members of the health team. They express confidence in patient assessment and communication skills and report taking greater responsibility for their own learning. Clinical nursing staff comment that students are more accountable for patient care than traditional model student. Clinical staff report they are comfortable that the quality of care is upheld in the new approach and are pleased to see that students are advancing clinically. In addition, clinical staff members report that they became more aware of the curriculum and clinical education requirements.

In 2007, the University of Michigan School of Nursing (UMSN) and nursing services at the University of Michigan Health System (UMHS) formed a partnership called The Initiative for Excellence in Clinical Education, Scholarship and Practice. The partnership was designed to leverage the resources and expertise of both organizations to advance all three mission areas.

To begin the work, the partnership engaged in an extensive change process using Danemiller Tyson Associates' Whole-Scale Change [14]. Whole-Scale change is large organizational change that occurs through the power of microcosms, groups comprised of members who represent and bring a perspective of every level and area of their organization, and nonmembers who represent those who rely on their organization to meet their needs. The assumption is that these stakeholders collectively have knowledge of the work of the whole system and their wisdom can bring about a new identity for how the organization will function in the future. For our two partnering organizations, stakeholders (**n** = 120) included members from all levels of staff nurses and nurse administrators, all levels of faculty including administrators and graduate student nursing instructors, nursing and medical students, physicians, patients, financial and human resource representation, and the vice-chair of the nurses' collective bargaining unit at UMHS. At a two-day retreat called an Event [14], stakeholders were placed in small groups and asked to define the current state of our working relationship with respect to clinical education, practice, and scholarship, to envision a different future state for our relationship in these mission areas and to propose actions to move toward the future state. A facilitator skilled in conducting Whole-Scale change guided the work of participants. From the considerable data generated throughout discussions, six major themes emerged: Teams, Innovative Strategies, Shared Vision, Physical Facilities, Human Resource Development, and Outcomes. A six-member Task Force with representation from both organizations was charged to elaborate each theme with actions culled from the larger dataset generated at the Event. These themes and actions subsequently would serve as the foundation for further work on each of the three mission areas—clinical education, practice, and scholarship.

Developing a clinical education model became the first priority for our work. By embedding students in the clinical setting with faculty who have clinical and research expertise education becomes the clinical bridge that anchors and connects all three mission areas. This link is critical both to advancing practice and solving clinical problems to improve patient outcomes. The purpose of this article is to describe the first phase of our collaboration, the development of the Clinical Education Model, with attention given to the recommendations put forward by the IOM, RWJF, and others.

## 2. The Clinical Education Model

A three-member Project Team consisting of two faculty from the UMSN and one Health System nursing director was charged to use the themes from the Event to develop a new clinical education model that was vetted and ready for pilot implementation in one year. In developing the new model, it was explicitly stated that the proposed model must align with values and goals of each organization and with current economic resources, that existing contractual and other legal and regulatory requirements must be honored, and that the model must align with the current undergraduate course curriculum. In addition, the new model was to reflect recommendations of organizations calling for change in how nurses were educated in order to better prepare them for current health care environments. An Advisory Committee to the Project Team was formed comprised of members from both organizations including the Dean, the CNO of the Health System, and students. The role of the Advisory Committee was to provide advice, consultation, feedback, and recommendations throughout development of the model and plan for pilot implementation.

That this is a true partnership between our organizations was demonstrated as the Project Team consulted with various faculty, students, and clinical partners in the Health System including Clinical Nurse Specialists, Educational Nurse Coordinators, Supervisors, and staff nurses for suggestions and endorsement throughout development of the model. Feedback from these stakeholders was instrumental in revising ongoing work. Circling back allowed stakeholders to see how their suggestions and concerns were handled. This approach to the work also permitted participants in the process to develop relationships over time and increase knowledge of Health System units, patient populations, nursing practice, student instruction, and the curriculum. Over the year of model development, 18 presentations and interactive sessions were held with various stakeholder groups. Final vetting occurred with both organizations including the nurses' collective bargaining unit in the Health System. The new model consists of five components: Clinical Unit Clusters, Clinical Teams, Student Skill Development, Clinical Immersion, and Student Portfolio.

### 2.1. Clinical Unit Clusters

**Figure 1:**
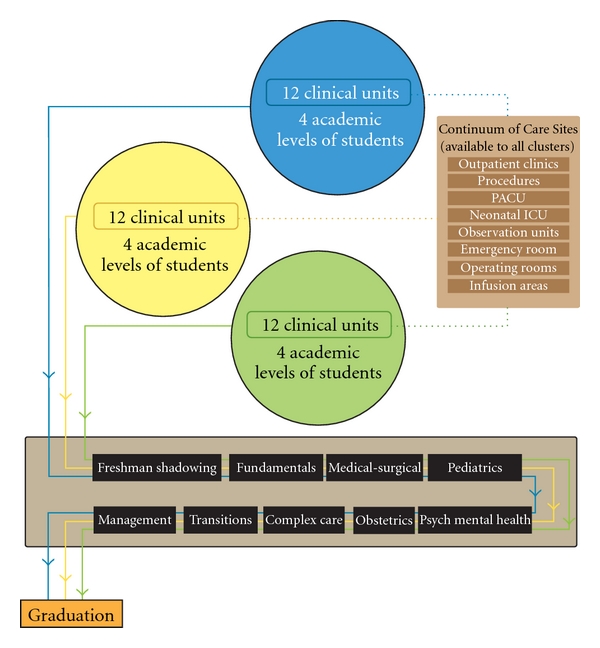
Clinical clusters, continuum of care, undergraduate clinical courses.

Maximizing UMSN student placement capacity at UMHS was highly desired as the environment provides strong evidence-based practice and quality of care is paramount. At the time of model development, many UMSN students received clinical education in multiple hospitals and agencies. In the new model at UMHS, all 36 inpatient clinical units, comprised of adult acute care, pediatrics, ICUs, and specialty services, were used to construct three multiunit clusters (see Figure 1) comprising key components of students' clinical requirements. Each cluster (Blue, Yellow, Green) includes students of all academic levels. Students organized in these clusters progress through undergraduate clinical courses over their four years of study. Continuums of Care areas available to all clusters are clinical areas in addition to inpatient units that provide care and services to patients as needed. Maximizing the use of these additional clinical areas affords students opportunities to participate in and follow patient care throughout the health system as they learn about nurses' roles in a variety of settings and reduces the need for placements at external agencies. In the current model, a few sections of obstetrical nursing and psychiatric nursing continue to be external due to current limited capacity of these services at UMHS. Community Health Nursing continues to be community based. 

Upon admission to the School of Nursing in the freshman year, students are assigned to one cluster and will have diverse clinical experience designed to meet all clinical course objectives across the four years of the curriculum. Students who transfer from community colleges at the junior level are entered into clusters when admitted. The assignment to clusters familiarizes students with a defined set of units, their operations, and the work of nurses as the context for clinical learning [2]. In addition, the clinical cluster approach creates cluster identity among faculty, staff, and students thereby enhancing relationships among these individuals in such a way that both education and patient care can benefit. Cluster unit familiarity has high potential to increase identification of clinical problems for further study both within and across units in the cluster. Health System Clinical Unit Profiles, descriptions of each clinical unit that include common patient populations, diagnoses, medications, and treatments, are given to students for each unit in their cluster. Profiles provide background information which orients students to the cluster of units to which they are assigned.

### 2.2. Clinical Team

In the model, a clinical team (see Table 1) is the relational structure between faculty, Clinical Mentor, Clinical Resources, and students in which clinical education of the students occurs. Five roles are described for each Clinical Team: (1) Faculty of Record, (2) Clinical Faculty, (3) Clinical Resources, comprised of current unit-based clinical leaders known as Clinical Nurse Manager, Clinical Nurse Supervisor, Clinical Nurse Specialist, and Educational Nurse Coordinator who serve as sources of information about patient care issues on an on-need basis, (4) Clinical Mentor, and (5) Student whose responsibility is to work closely with the Clinical Mentor and Clinical Faculty in providing patient care while progressing in a learning environment. Students are involved in the Mentor's entire patient assignment over the course of the shift. Thus, the student is an integral team member and not a “guest” in the patient care environment [2]. Table 1 provides role descriptions.

All members of a clinical team are expected to develop strong relationships and function as an integrated group rather than through a hierarchical structure of accountability. Clinical Faculty are matched as much as possible where their clinical skills and knowledge are compatible with unit patient populations as they are responsible not only for clinical education but for participation in scholarship and practice development on these units as well. They may provide direct instruction of the student but are more likely to facilitate the student's learning through work with the Clinical Mentors. Importantly, they model appropriate teaching strategies, clinical expert practice, and an understanding of evidence-based practice and its application. The Clinical Mentor assumes a direct role in student education while using Clinical Faculty for guidance in conducting the student's experience.

**Figure 2:**
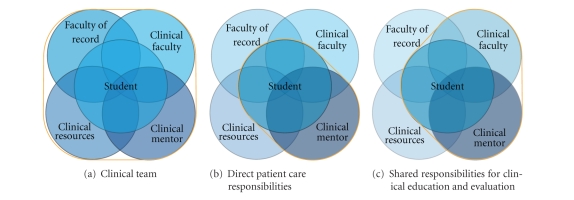
Clinical team.

Figure 2(a) shows the relational patterns among all members of the team.  Figure 2(b) reflects a shift in teaching responsibility as Clinical Mentors work closely with students while they engage in clinical care.  Figure 2(c) shows the direct role that Clinical Faculty bring to the partnership including oversight of student assignment and some direct instruction of the student, modeling teaching strategies to the Mentor, ensuring that course objectives are being met, and participating with the Mentor in assessing student performance. These figures demonstrate, at the unit level, the partnership that was envisioned at the Whole-Scale Change event. 

In addition to preparation for the role of Clinical Mentor, a document developed by nursing staff and clinical unit leaders, with added input from the Project Team, specifies essential characteristics guiding the transition into the Mentor's new responsibilities. This document charts the growing responsibilities of Mentors as they mature in the role. Members of the clinical team together provide a rich environment in which to nurture and support student growth in clinical competence.

### 2.3. Student Skill Development

In reframing the student's role as a team member, new approaches were needed for skill preparation for clinical courses. Faculty were aware from prior nursing staff feedback that increased skill preparation for students was necessary for successful student integration into the clinical team. Following an intensive workshop for faculty and clinical partners on simulation use in student education, new simulations were designed and embedded in clinical courses to improve clinical reasoning in the delivery of patient care. Planned laboratory time provides for practice and evaluation of student competence in relevant skills. Successful completion increases the students' ability to participate in patient care as an integral member of the clinical team.

### 2.4. Clinical Immersion

Increasing the number of hours devoted to clinical practice is important for gaining competence and feeling confident in providing care. Over time, nursing curricula have included more didactic content and fewer clinical hours. In addition, student clinical assignments often have been limited to the care of one or two patients not only early in clinical experience but throughout the entire curriculum [2]. Current clinical environments demand that the nurse be able to manage a larger assignment while providing exemplary evidence-based care. The clinical model incorporates intensive clinical experiences in which students are fully engaged in a practice environment providing care to patients and families. 

### 2.5. Portfolio

A paper portfolio consisting of course descriptions, Clinical Unit Profiles, Student Skills Checklists for both completed skills and those to be achieved, and Midterm and Final Student Clinical Evaluation Forms is given to students. A Clinical Mentor Feedback Form to serve as a communication tool between the Mentor and student about the student's performance and to provide written documentation for Clinical Faculty is also included. Clinical course materials for subsequent courses are added as the student progresses in the curriculum. The portfolio is designed to be utilized by all Clinical Team members to facilitate student learning and performance. Transition to an electronic portfolio is to be determined by the patterns of use, value of current materials, and compatibility with UMHS systems.

## 3. The Clinical Pilot

The clinical education model was pilot tested in the junior year of the generic undergraduate program from September, 2008 through April, 2009 when the academic term ended. Since it was not reasonable at the outset to test the model for all four years of the curriculum, the decision was that the pilot would include only students in the junior year when they were enrolled in specialty clinical courses.

 Half the students (*n* = 31) enrolled concurrently in medical-surgical and pediatric nursing courses participated during the fall term pilot. They continued in the pilot during the winter term when enrolled in psychiatric and obstetrical nursing courses. An additional 30 students who completed the psychiatric and obstetrical nursing courses in the fall joined the pilot for the first time in the winter term for a one-term pilot experience. By the end of the winter term, half the junior class (*n* = 61) had experience in the new clinical education model. The selection of clinical units to participate in the pilot was decided following conversation with unit leaders and staff. The decision was that highly enthusiastic units would be asked to participate as the likelihood for demonstrating success of the clinical model would occur with units that were more positive about change and willing to work to enhance the success of our partnership. In the fall term pilot, three medical-surgical units and one large pediatric unit participated. During the winter term, the large women's birthing unit and one adult and one child psychiatric unit were added. All units were in the Blue Cluster.

Orientation and training to the model and the operation of its components was developed for all pilot participants including clinical leaders, staff who would become Clinical Mentors, Clinical Faculty, and pilot students. Each session was three hours. Introductory time was allotted to meet each other and hear the vision of the new partnership from the Dean and the CNO. Planned exercises were designed to prepare participants to successfully inhabit new roles and responsibilities, and to increase understanding of communication strategies in the patient care settings. To create an identity for The Initiative, a logo and color scheme was designed and used for all materials posted in common areas, distributed materials, presentations, and portfolio covers. All Clinical Faculty attending the Orientation and Training had been teaching in the traditional clinical program and would be continuing in the new education model. Nursing leaders and nursing staff who attended had worked previously with students from UMSN.

A series of upfront skills sessions, relevant for the clinical courses in which students were enrolled, was designed and took place during the first month of each semester before clinical experience began (see Table 2). These skills and scenarios were in addition to those covered in the lecture portion of the clinical courses. Faculty and clinicians from UMHS presented material and demonstrated skills including use of high-fidelity mannequins. Skills sessions were accompanied by scenarios of clinical events that could occur in patient care areas that required intervention. Practice opportunities for students followed and included practice of the skill as well as clinical reasoning exercises to support decisions made during skill demonstration. Materials were made available for student use during and following demonstration. During this month, students attended class lectures and completed course assignments. Their clinical experience on the units began in the fifth week of the term. They were paired with a unit Clinical Mentor and began their role as a member of the Clinical Team.

The clinical immersion experience (Table 2) consisted of two consecutive days per week alternating clinical specialties each week (i.e., medical-surgical nursing the first week and pediatric nursing the second) for five weeks followed by three weeks of four consecutive clinical days. The remaining week of the term reverted back to a two-day experience to accommodate the academic calendar. In Winter term, the same plan was followed for ongoing pilot students now enrolled in obstetrical and psychiatric nursing. New students to the pilot in Winter term followed the same clinical schedule as their peers in Fall term. The immersion was intended to actively engage students in clinical work, to increase competence, and to enhance their understanding of the nurse's role in managing holistic change in patients over time. Students were expected to be fully involved in the Mentor's entire patient assignment over the course of the shift. Pilot students used the clinical portfolio to guide clinical work and collaborate with Mentors on assessment of performance.

Pilot and nonpilot students were together for the didactic portion of each of the clinical courses. Aside from shared class attendance nonpilot junior students followed the traditional model of clinical education. They began their clinical experiences at the start of the term and did not participate in the upfront skills sessions. They followed the typical student plan for clinical experiences of two days per week alternating clinical specialties each day (i.e., medical-surgical nursing the first day and pediatric nursing the second) across the entire term. The Clinical Faculty member on the unit was largely responsible for selecting patient assignments, monitoring student performance, and assessing performance. Nonpilot students were not part of a clinical team, and they did not use a student portfolio. Clinical Faculty solicited input from staff about students' performance and provided this feedback to students and used it as needed in written evaluations.

Throughout the pilot of the clinical model, Project Team members visited clinical units to monitor the learning environment. Discussion occurred with Clinical Faculty, Mentors, and students to solve problems, answer questions about team functions, or work one on one with team members to support their respective roles in the Clinical Team. Occasionally, clinical unit meetings were held to further explain the model and respond to questions.

## 4. Evaluation Methods

Clinical Faculty, Clinical Mentors, Clinical Resources, and pilot students participated in clinical model evaluation. Nonpilot (Traditional students) were not asked about their experiences in the traditional model or their thoughts about how not having the pilot opportunity influenced their perception of their education.

Feedback regarding the clinical cluster arrangement was gathered during meetings of unit representatives from each cluster group—Clinical Resources, Mentors, and Clinical Faculty. Clinical Cluster meetings were typically held twice a year, once per term, for discussion and confirmation of upcoming clinical placements. Data from two discussion sessions with Clinical Faculty, Clinical Leaders, and Clinical Mentors (*N* = 23, Fall and 25 participants Winter) constituted the method for data collection during and following the pilot. On average, representation was approximately 4 representatives from each pilot unit including the Clinical Faculty member. The number of attendees per unit attending the discussions is acceptable given that time away from the unit needed to be negotiated in advance to cover staffing and students. Prior to the discussion sessions, attendees talked with others on their respective units to gather information in order to bring as many perspectives as possible to the discussions. Components of the clinical education model—Clinical Teams, student skills preparation, clinical immersion, and student portfolio guided discussions.

Focus groups with pilot students were designed to elicit information about each of the components of the clinical mode. Feedback was obtained at term's end (*N* = 25) for the Fall term pilot students (Medical-Surgical/Pediatric courses) and at the end of the Winter term from this same group of students (*N* = 25) now enrolled in Obstetrical/Psychiatric Nursing courses. Feedback from the one-term Winter pilot students (*N* = 24) in Medical-Surgical/Pediatric Nursing courses was also obtained at the end of the term. The one-hour focus groups were led by a skilled facilitator who was unknown to the students thus reducing worry about being candid. Faculty were not present in order to maintain an unbiased and nonthreatening environment. This number exceeds that typically recommended for sessions [15] but did not restrict students from expressing, concurring, or differing with views expressed by peers. The facilitator summarized information after each discussion of a clinical model component to clarify that the information was an accurate representation of views. Changes to the information were based on student input and agreement. Each session was taped, session notes were taken by a recorder, and a summary report was generated by the facilitator. 

## 5. Key Findings

### 5.1. Clinical Clusters

The clinical cluster concept for student education was new to faculty and clinical staff. These members supported the concept of clinical clusters and agreed to continue defining working relationships both within and across clusters. One immediate and very positive outcome of work across clusters was a more efficient clinical placement process of UMSN students.

### 5.2. Clinical Teams

During discussion sessions, faculty described needing to adjust their role from full responsibility for student education to shared responsibility with Mentors. This change required modeling best education practices as well as facilitating Mentors in their new role in the clinical team. Faculty also reported that their working relationships with unit nurses improved. Initially, some Mentors were tentative about their new role and engaged students less fully in the team (see Table 3) until, with faculty and unit leadership support, their comfort level increased. The experience level of Mentor seemed not to affect ability to function in the role. Clinical Faculty and Clinical Mentors both indicated that students benefitted from being a member of a clinical team and they soon adjusted to their new role in the team. Mentors, in conversations with faculty reported using more clinical reasoning strategies with students, and those who had worked with UMSN students previously indicated it was their impression that students' clinical performance was accelerated beyond previous cohorts of students. Mentors commented that their own practices were enhanced by the new clinical education model.

Focus group discussions with students (see Table 4) revealed that they enjoyed working in the clinical team and most became fully integrated over the term. Being fully integrated, according to students, meant that they were involved in all aspects of their Mentor's assignment. Students agreed that through experience as a team member they became more skillful in organizing work, communicating with others, practicing with greater independence, and identifying and requesting learning opportunities. Students also reported that they benefitted from frequent feedback from Mentors during the day about how the day's work was progressing. They acknowledged that some Mentors were less eager to include students in their assignments.

### 5.3. Skills Preparation

Overall, the upfront presentation of clinical skills did not fare as well as expected. Both Clinical Faculty and Mentors agreed that skill preparation should be increased throughout the curriculum with unit-based skills emphasized during clinical conference time on the unit. Mentors added that being able to receive and give report to others at shift changes is an essential skill to be included. Students maintained during focus group discussions that time were not used well, sessions were long and with limited practice, and unit-based skills they felt were necessary were not included. During Winter term, the addition of unit-specific skills to clinical unit orientation and clinical conferences was seen to facilitate learning and skill performance by students, especially when accompanied by clinical scenarios.

### 5.4. Clinical Immersion

As shown in Table 3, Clinical Faculty and Mentors agreed that two consecutive clinical days and later four consecutive clinical days increased student competence over the term. Toward the end of the term, during the four consecutive clinical day experience, faculty reported in that students' time-management skills increased noticeably from the beginning of the term. They also noticed that students seemed tired from the extended clinical time. Students (see Table 4) reported having a better understanding of patients' experiences and care with increased time and continuity of patient assignments. In addition, they reported their clinical capabilities had increased from the start of the term and patient relationships were strengthened over the course of the immersion experience.

### 5.5. Portfolio

The utility of the portfolio as a whole was not commented on by Clinical Faculty, Clinical Mentors, or students. However, one document, the Clinical Mentor Feedback Form used jointly by the student and Mentor to assess and summarize the student's clinical day, was singled out and by all groups (Tables 3 and 4). Mentors found the 1–4 rating of student performance hard to use and tended to rate students highly as they were reluctant to use the lower range that might discourage students. They indicated that discussing expectations with faculty about student performance would help with using the scale wisely. Both Clinical Faculty and Mentors agreed that written comments were more informative than the rating scale. Students preferred written comments and recommended that the form provide more space for these as they are more informative than numerical ratings.

## 6. Discussion

The vision for a new clinical education model has become a reality and the pilot has shown its potential as well as pointed out areas for improvement. The partnership envisioned by participants in whole scale was demonstrated at every level of model development, in planning for pilot implementation, and in carrying out the pilot. The enthusiasm of units willing to participate in the pilot ensured that every effort would be placed in making the model work. Findings from the pilot revealed the components of the model that were working well and those that required additional effort. Clustering students in a designated set of units for the entire curriculum was endorsed. Projected calculations showed that the new centralized student placement process can accommodate student placement in other years of the curriculum aside from the junior year including Freshman (shadowing experience), Sophomore, and Senior students who need or select placements in our health system. The full potential of clusters supporting education of students housed within them, and in promoting clinical research, continues to be developed.

The clinical team approach to educating UMSN students is new. The traditional faculty role of primary supervision of students changed to that of facilitating and supporting the Mentor who, in turn, took greater responsibility for student learning. Some faculty, early on, were not convinced that this shift in responsibility was appropriate. However, as they participated in the clinical team, they adapted to the new role that included an increase in role-modeling instructional strategies and support for Mentor activities. Clinical Mentors varied in how quickly they accommodated to their revised role in student education, most assumed the new role fairly quickly and easily. Annoyance was expressed by some Mentors who indicated that education of students took too much time and tended to be a burden. With support from unit leadership, the Project Team, and faculty, most became experienced team members. In our model students, as part of a designated clinical team, participate in the Mentor's entire assignment. The relationship among team members provides a rich environment for student learning. Students in the pilot stated that clinical experiences enhanced their learning and that working with the Mentor and other unit leaders gave them a strong sense of contributing to the work of the team while learning. Additional support that the team approach to clinical education worked is that our findings were similar to those found using the DEU. For example, students in our model, like those in the DEU approach, felt they were included in the clinical team, supported by Mentors, more confident in providing patient care and able to assume more responsibility for their own learning [9, 10, 12, 13].

The clinical immersion was rated highly by students. They benefitted from continuity of patient care, became more confident of their capabilities, and began to experience more fully the work of the staff nurse. Student skill preparation has been changed, based on student and faculty input, to include greater emphasis on clinical unit relevant skills in addition to general skills taught in conjunction with courses. Preliminary evidence in the pilot showed that this approach is working.

Although the clinical education model was pilot tested with half of the Junior students, the pilot was not conducted as an experimental study to compare the views and outcomes between pilot and nonpilot students. As a result, nonpilot students were not asked to provide their views about being a student in the traditional program while peers were receiving a different approach to clinical education. Information from nonpilot students may have been useful to understanding their thoughts about the merits of the different approaches, or how being treated differently from their peers influenced their views about the quality of education they were receiving. Comments expressed by nonpilot students in informal venues revealed a mix of responses. Some were angry that they were not asked how things were going for them clinically. Some expressed relief from not having to attend the intense skill preparation sessions or complete the four-day clinical immersion which they stated seemed exhausting for their classmates. Some also mentioned that having fewer clinical obligations at the end of the term left them more time to study. A number commented that they were happy not to be part of an “experiment” and felt comfort in knowing that the traditional curriculum was known to faculty and nurses in UMHS with whom they worked. Although nonpilot student comments may have been informative, they would not have changed the decision to move forward with the new clinical education model. The clinical education model is part of a larger vision of the partnership between the UMSN and UMHS about how we will work together in advancing the missions of excellence in education, research, and practice, with education as the anchor for advancing the other two missions. The new education model is consistent with calls from the IOM and RWJF to restructure education programs to be consistent with 21st century health systems [4, 5], and to prepare nurses for new responsibilities in rapidly changing health care environments [2]. Partnerships and teams are emphasized by these organizations as being critical to improving health care.

Full implementation of the model for all junior students and to all 36 clinical units in UMHS in 2009 followed the pilot. Some changes in the model have occurred. Because it became difficult to structure faculty workload to accommodate both the two-day and four-day clinical immersion over the term, a different approach to clinical immersion was taken. The undergraduate clinical curriculum was revised and approved to add and progressively increase clinical hours from freshman through senior year culminating in a three-day per week capstone clinical practicum over the term supported by seminar. To ensure skill readiness for beginning clinical work, students must be checked off each term on skills they learned in clinical courses the previous term before they can begin the new term's clinical experience. Failure to pass requires repeat testing that may delay the start of clinical work.

Last, and very important, two new clinical track faculty roles are now in place. Clinical Cluster Leads are faculty educators who provide full-time leadership and education to faculty, staff, and students in a cluster. Responsibilities include supporting coordination of clinical placements in the cluster, facilitating faculty in clinical education and practice, and engaging in and leading others in the conduct of clinical research. Clinical Educators are clinical faculty who, in addition to providing supervision and education of students in the clinical setting, will build strong partnerships with Clinical Mentors and through these partnerships conduct scholarly work designed to improve patient outcomes and advance nursing practice. The Clinical Educator now becomes the Clinical Faculty in the model. These two roles require doctoral preparation. Clinical Cluster Leads and Clinical Educators are embedded full time in the clinical setting. These faculty and students comprise the structure of the clinical bridge that anchors and connects the mission areas all three mission areas.

## 7. Conclusion

The vision for a new clinical education model has become a reality and the partnership between UMSN and the UMHS became stronger as the model was developed and implemented in the Health System. It continues to be a work in progress with the goals of preparing nurses to provide state-of-the-art care to patients and families, adapt to new approaches in rapidly changing health care environments, use evidence to ground practice, and participate in exploring nurse sensitive solutions to clinical problems.

## Figures and Tables

**Table 1:** Clinical team teaching roles.

Team member	Role description
Faculty of record	Member who facilitates the delivery of course materials and translates course requirements to the Clinical Team in addition to other responsibilities associated with the didactic portion of the course
Clinical faculty	Member who oversees delivery of the clinical practice experience for students to meet clinical course objectives
Clinical resource	Unit nursing leadership member who supports learning at the point of service through integration of faculty and students into unit operations
Clinical Mentor	Staff nurse at UMHS who, consistent with scope of practice of the Registered Nurse, models the professional nurse role and participates in the clinical education of students
Student	U of M nursing student who participates fully in clinical care through integrated membership in the clinical team, engages in the educational activities designed to achieve course outcomes and to build the foundation for nursing practice, and contributes to patient care using the skills for which the student has been determined competent

**Table 2:** Students clinical immersion schedule.

Week	1	2	3	4	5	6	7	8	9	10$\hat{\;}$	11$\hat{\;}$ $\hat{\;}$	12	13	14
Days	0*	0	0	0	MT	**	MT	MT	MT	MT	MT/WTh	MT/WTh	MT/WTh	MT

Course(s)	0	0	0	0	P°		MS°	P	MS	P	MS/P	MS/P	MS/P	MS
	0	0	0	0	MS		P	MS	P	MS	P/MS	P/MS	P/MS	P

Legend: ^*^*^: classes completed week 10. ^*^* *^*^: week 11 the 4 day clinical immersion. *: no clinical/class day only. **: MT study break no clinical/class held. °: Pediatrics or medical/surgical nursing.

**Table 3:** Pilot faculty and clinical staff discussion group comments.

Fall Term 2008 (Medical Surgical and Pediatric Courses) *N* = 23	

Clinical Teams	
(i) RN staff varies in ability to function in Clinical Mentor role regardless of attending orientation or training	
(ii) Over the term positive relationships developed between students and Clinical Mentors	
(iii) If choice needs to be made between student continuity with a patient or a Clinical Mentor, a consistent patient assignment was preferred	
(iv) Adjustment to new collaborative role was achieved and viewed positively	
Skills Preparation	
(i) Skills sessions did not make an appreciable difference in students transition to clinical practice	
(ii) More direct unit based skills training and evaluation of student competency needed	
(iii) Expectations of student clinical performance should be consistent between Faculty and Clinical Mentors	
Clinical Immersion	
(i) By terms end, two consecutive days of clinical practice was viewed as a strength	
(ii) Advantage of 4 day clinical immersion was found in time-management skills and continuity of patient care	
(iii) Compressed class time seen as tiring for students	
Portfolio: Clinical Mentor Clinical Feedback Form	
(i) Portfolio became useful once student had more clinical experience	
(ii) Clinical Mentors were inclined to rate students highly while not addressing areas for improvement	
(iii) Written comments were more helpful then rating scale based evaluation	

Winter 2009 (Medical Surgical, Pediatric, Obstetric and Psych Courses) *N* = 25	

Clinical Teams	
(i) New RN's were seen as strong collaborators with students and Faculty.	
(ii) Clinical Mentor confidence in their own abilities improved over course of term	
(iii) Some concern regarding slower identification of lower functioning students when paired with multiple Clinical Mentors. Consistency of pairing a priority for some students	
Skills Preparation	
(i) Faculty as well as students need to be knowledgeable of unit based clinical skills	
(ii) How to give and receive shift report identified as a valuable skill for students to learn	
Clinical Immersion	
(i) Units with highly complex patient populations experience Clinical Mentor and student fatigue	
(ii) Collaborating with Clinical Mentor on full patient assignment improved student confidence	
Portfolio: Clinical Mentor Clinical Feedback Form	
(i) Training in how to give and receive feedback needed for both Clinical Mentors and students	
(ii) Clinical Mentors need support of Faculty to openly discuss expectations with students	
(iii) Students need encouragement to converse with Clinical Mentors about expectations and performance	
(iv) Clinical Mentor feedback form requires revision to capture more dynamic and useful feedback of student performance	

**Table 4:** Pilot student focus group comments.

Fall 2008 (Medical Surgical and Pediatric Courses) *N* = 25	

Clinical Teams	
(i) Described themselves as fully integrated into team over the term	
(ii) Increasingly communicated with health care provider	
(iii) Better understanding of the roles of Clinical Resources	
Skills Preparation	
(i) Skills preparation less than useful	
(ii) Requested more unit-based skills	
Clinical Immersion	
(i) Increasingly more comfortable and confident in clinical practice	
(ii) Continuity in clinical care was seen as a plus and team relationships grew	
Portfolio: Clinical Mentor Clinical Feedback Form	
(i) Further orientation and training required to achieve full benefit of portfolio use	
(ii) Written comments perceived to have more value than ratings scale	

Winter 2009 (Medical Surgical, Pediatric, Obstetric and Psych Courses) *N* = 49	

Clinical Teams	
(i) Experienced Mentors were perceived as more comfortable in the role of clinical educator and with integrating students into the clinical team	
(ii) Practiced over the term with greater independence	
(iii) Mentor feedback beneficial, post care debriefing sessions viewed positively	
(iv) Some RNs less willing to engage students in clinical practice	
Skills Preparation	
(i) Skill learning/performance increased when skills tied to unit based clinical practice	
(ii) Skills reenforced when unit based scenarios used in clinical conference	
Clinical Immersion	
(i) Provided realistic look at life as a nurse	
(ii) Improved patient relationships and understanding of experience from patients' perspectives	
(iii) Being proactive in seeking opportunities added a positive effect on overall experience	
Portfolio: Clinical Mentor Clinical Feedback Form	
(i) Recommended defined comment section on student performance, that is, safety, clinical skills, communication	
(ii) Mentor familiarity and preparedness affecting value of feedback	
(iii) Student involvement in evaluation increased understanding of clinical performance	

## References

[1] American Nurses Association (2002). Nursing’s agenda for the future: a call to the nation. http://www.ana.org/naf/Plan.pdf.

[2] Benner P, Sutphen M, Leonard V, Day L (2010). *Educating Nurses: A Call for Radical Transformation*.

[3] National League for Nursing (2004). Position statement on innovation in nursing education: a call to reform. *Nursing Education Perspectives*.

[4] Robert Wood Johnson Foundation (2002). Health care’s human crisis: the American nursing shortage. http://www.rwjf.org/files/newsroom/NursingReport.pdf.

[5] Institute of Medicine (2001). *Crossing the Quality Chasm: A New Health System for the 21st Century*.

[6] Institute of Medicine (US) (2010). *Initiative on the Future of Nursing: Leading Change, Advancing Health*.

[7] Edgecombe K, Wotton K, Gonda J, Mason P (1999). Dedicated education units: 1. a new concept for clinical teaching and learning. *Contemporary Nurse*.

[8] Barnett T, Cross M, Shahwan-Akl L, Jacob E (2010). The evaluation of a successful collaborative education model to expand student clinical placements. *Nurse Education in Practice*.

[9] Haas BK, Deardorff KU, Klotz L, Baker B, Coleman J, DeWitt A (2002). Creating a collaborative partnership between academia and service. *The Journal of nursing education*.

[10] Moscato SR, Miller J, Logsdon K, Weinberg S, Chorpenning L (2007). Dedicated education unit: an innovative clinical partner education model. *Nursing Outlook*.

[11] Murray TA, Crain C, Meyer GA, McDonough ME, Schweiss DM (2010). Building bridges: an innovative academic-service partnership. *Nursing Outlook*.

[12] Ranse K, Grealish L (2007). Nursing students’ perceptions of learning in the clinical setting of the Dedicated Education Unit. *Journal of Advanced Nursing*.

[13] Wotton K, Gonda J (2004). Clinician and student evaluation of a collaborative clinical teaching model. *Nurse Education in Practice*.

[14] Dannemiller Tyson Associates (2000). *Whole-Scale Change Unleashing the Magic in Organizations*.

[15] Burns N, Grove SK (2007). *Understanding Nursing Research: Building an Evidence Based Practice*.

